# Mixing and matching mitochondrial aminoacyl synthetases and their tRNAs: a new way to treat respiratory chain disorders?

**DOI:** 10.1002/emmm.201303586

**Published:** 2014-01-29

**Authors:** Henna Tyynismaa, Eric A Schon

**Affiliations:** 1 Research Programs Unit, Molecular Neurology, Biomedicum HelsinkiHelsinki, Finland; 2 Department of Medical Genetics, Haartman Institute, University of HelsinkiHelsinki, Finland; 3 Department of Neurology, Columbia University Medical CenterNew York, NY, USA; 4 Department of Genetics and Development, Columbia University Medical CenterNew York, NY, USA

## Abstract

Mutations in mitochondrial DNA are an important cause of human disease and from a therapeutic standpoint, these disorders are currently untreatable. New studies now show that a non-cognate mitochondrial aminoacyl tRNA synthetase can overcome the respiratory defect caused by an mt-tRNA mutation and that the isolated carboxy-terminal domain of human mt-leucyl tRNA synthetase can ameliorate the pathologic phenotype.

See also: **HT Hornig-Do *et al*** (February 2014) and **E Perli *et al*** (February 2014)

Mutations in mitochondrial DNA (mtDNA) – a tiny circle encoding 13 polypeptides, 2 rRNAs, and 22 tRNAs (Fig 1) – are an important cause of human disease. The tRNA genes cover <10% of the total 16.6-kb of human mtDNA, yet about half of the known pathogenic mutations are found in the tRNAs. Even though most mutations in tRNAs affect mitochondrial translation, the resulting clinical phenotypes are remarkably heterogeneous. From a therapeutic standpoint, essentially all of these disorders are currently untreatable, in part because attempts to correct mutations in mtDNA in a stable and heritable way have not yet succeeded. Thus, creative alternative means to influence the onset and progression of mtDNA-based diseases are being investigated.

**Figure 1:**
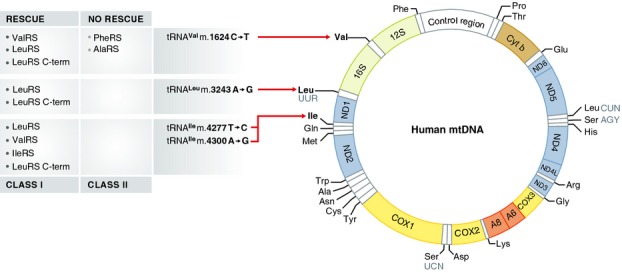
The human mitochondrial DNA 16.6-kb genome encodes 7 subunits of complex I (ND1, 2, 3, 4L, 4, 5, and 6), 1 subunit of complex III (Cyt b), 3 subunits of complex IV (COX1, 2, and 3), and 2 subunits of complex V (A6 and 8), as well as 2 rRNAs (12S and 16S) and 22 tRNAs (3-letter notation).

“Even though most mutations in tRNAs affect mitochondrial translation, the resulting clinical phenotypes are remarkably heterogeneous.”

Pathogenic mutations in mt-tRNAs typically interfere directly with cellular ATP production, because the tRNAs are part of the organellar translation system devoted to the synthesis of the 13 subunits of the oxidative phosphorylation (OXPHOS) complexes that are encoded by mtDNA. Except for the two ribosomal RNAs (also mtDNA-encoded), all the other factors necessary for mitochondrial protein synthesis are encoded by nuclear genes and are imported into mitochondria from the cytoplasm. These include the protein subunits of the mitoribosome, translation elongation factors, and the aminoacyl-tRNA synthetases (aaRSs). For most amino acids, the cognate aaRS for each mitochondrial tRNA is distinct from the counterpart tRNA synthetase that functions in the cytoplasm.

Although aaRSs have the basic housekeeping function of charging tRNAs with their cognate amino acids in the initiation step of protein synthesis, recent studies have revealed unexpectedly versatile “moonlighting” functions for many of the cytoplasmic synthetases (Guo & Schimmel, 2013), in pathways ranging from angiogenesis to inflammation and apoptosis. Many of these functions are performed by added domains in the aaRS that are dispensable for aminoacylation. For example, a C-terminal “added” domain, UNE-S, in the cytoplasmic SerRS, is essential for vascular development through directing SerRS to the nucleus, where it regulates VEGF-A transcription (Xu *et al*, 2012). Such domains with “non-canonical” functions have not been described previously for mammalian mitochondrial aaRS's, but a domain of the mitochondrial leucyl-tRNA synthetase (mtLeuRS) from *S. cerevisiae* has been shown to have group I intron splicing activity (Labouesse, 1990). Importantly for mitochondrial tRNA disease, this mtLeuRS domain was shown to rescue mutants in yeast mt-tRNAs equivalent to pathological mutations in the corresponding human tRNA (Francisci *et al*, 2011). In addition, several examples exist where the expression level of the cognate aaRS modulates the effect of the mitochondrial tRNA mutation, in both yeast and human cell models: for example, mtLeuRS suppressed a tRNA^Leu^ mutation in human cells (Park *et al*, 2008) and a female with a pathogenic tRNA^Ile^ mutation remained clinically unaffected because she had a naturally higher expression level of mtIleRS (Perli *et al*, 2012). Another recent demonstration of how aaRSs could influence the penetrance of mitochondrial diseases is the work by Meiklejohn *et al* (2013) showing incompatibility of mtTyrRS and mt-tRNA^Tyr^ genotypes modulating mitochondrial protein synthesis in *Drosophila*. It should also be noted that the first reported mitochondrial tRNA rescue was not by an aaRS, but by a naturally-occurring structurally modified tRNA^Leu(CUN)^ that suppressed a mutation in tRNA^Leu(UUR)^ (El Meziane *et al* 1998).

“Considering that there are no rational treatments for mitochondrial tRNA diseases, a universal suppressor molecule for all tRNA mutations would be an outstanding advance, given, of course, all the caveats that attend gene therapy-based approaches to treating human disease.”

Inspired by the above-mentioned findings, and as reported in this issue of EMBO Molecular Medicine, Hornig-Do *et al*^2014^ and Perli *et al* have now tested the ability of a human C-terminal mtLeuRS fragment to rescue pathogenic tRNA mutations in human patient-derived cytoplasmic hybrid (cybrid) cell models. Their results show that the C-terminal mtLeuRS can be imported in significant amounts into mitochondria (although it lacks a typical mitochondrial localization signal) and is then sufficient to ameliorate the bioenergetic defects of the cybrids. Importantly, rescue by the C-terminal mtLeuRS was demonstrated for the m.3243A>G mutation in tRNA^Leu(UUR)^, which is associated with MELAS (mitochondrial encephalomyopathy, lactic acidosis, and stroke-like episodes), one of the most common, and devastating, mtDNA disorders (Perli *et al*, 2014).

While rescue of a tRNA mutation by its cognate aaRS, by whatever mechanism (see below), makes a certain amount of sense, what is more surprising is the finding that overexpression of an aaRS can ameliorate dysfunction caused by mutations in *non-cognate* tRNAs. The aaRSs are divided into two classes (I and II), based on the architecture of the active site (Ribas de Pouplana & Schimmel, 2001). Hornig-Do *et al*^2014^ provide evidence that the C-terminal mtLeuRS (Class I) binds to a number of mitochondrial tRNAs, but the rescue effect on mutations was demonstrated here only within Class I (Fig 1). The studies focusing on pathogenic mutations in tRNA^Ile^, tRNA^Leu^, and tRNA^Val^ and their cognate aaRSs showed interchangeability, but it should be noted that these Class I aaRSs are particularly closely related. The generalizability of the results for the other Class I aaRSs and for Class II aaRSs remains to be verified. Considering that there are no rational treatments for mitochondrial tRNA diseases, a universal suppressor molecule for all tRNA mutations would be an outstanding advance, given, of course, all the caveats that attend gene therapy-based approaches to treating human disease.

The ability of the C-terminal mtLeuRS to suppress functional defects caused by tRNA mutations is intriguing, but the mechanism of rescue is still controversial. Almost certainly it does not involve the aminoacylation domain of the aaRS, since that region was absent in the C-terminal fragment used by both groups to rescue function. Rather, the suspected RNA binding function of the C-terminal domain of yeast mtLeuRS (Labouesse, 1990) suggests that it functions by stabilizing the mutant tRNAs. Hornig-Do *et al*^2014^ show precisely that for the tRNA^Ile^ mutation that they studied. The steady-state levels of the uncharged mutant tRNA were significantly lower in the untreated cybrid line, and increased following the induction of mtLeuRS C-term expression, leading to enhanced mitochondrial translation, as expected. On the other hand, Perli *et al* saw no change in the levels of MELAS-mutant tRNA^Leu^ after mtLeuRS C-term overexpression, although we note that this tRNA was largely unstable to begin with. Taken together, these results indicate that the mechanism of rescue is more complicated than simple general stabilization of the tRNA, and depends on the specific tRNA and mutation in question. For instance, the differential post-transcriptional regulation of tRNA levels could be a factor (King & Attardi, 1993). Considering the diverse effects that the different tRNA mutations may have on tRNA function-for example, the lack of taurine modification at the anticodon wobble position in tRNA^Leu(UUR)^ in MELAS (Suzuki *et al*, 2002) – the C-term mtLeuRS may in fact aid the mutant tRNAs in multiple ways. Clearly, development of optimal treatment strategies will require a more precise understanding of the exact molecular mechanisms of rescue.

Among the necessary future work is to identify the minimal C-terminal fragment that still retains both mitochondrial localization and rescue ability, and to optimize the delivery method. Delivery of a short peptide could be ideal as a treatment, although obvious hurdles regarding pan-tissue delivery, stability, and potential toxicity present themselves. Even if sufficient amounts of the peptide were to enter the mitochondria, the remainder will stay in the cytoplasm and it is not yet clear whether it might interact with any of the cytoplasmic tRNAs and cause possible side effects. Unfortunately mouse models of targeted mt-tRNA point mutations are not available for testing, because of our inability to directly manipulate the mammalian mitochondrial genome in a stable and heritable way.

The importance of the mtLeuRS C-terminus in understanding the basic biology of mammalian mitochondria is also worth investigating further – for example, does it have an essential stabilizing function for some or all mitochondrial tRNAs through its RNA binding ability? Do other aaRS C-termini have a similar function? Do those from Class I and Class II aaRS's behave differently? Finally, mutations in many mitochondrial aaRSs themselves have recently been found to cause mitochondrial diseases (Konovalova & Tyynismaa, 2013). Perhaps the C-terminal mtLeuRS could improve the compromised tRNA stability in some of these patients as well.

In summary, therapeutic options for mitochondrial diseases are scarce and novel ideas for treatment may arise from unexpected directions. Detailed work to understand basic functions of mitochondria and their evolution are the key to many discoveries. The mtLeuRS peptide is still far away from the clinic, but its potential in treating mitochondrial tRNA diseases is clearly worthy of further investigation. The two papers reported here are showing us the way.
